# Prognosis of Coronary Atherosclerotic Burden in Non-Ischemic Dilated Cardiomyopathies

**DOI:** 10.3390/jcm10102183

**Published:** 2021-05-18

**Authors:** Marjorie Canu, Léa Margerit, Ismail Mekhdoul, Alexis Broisat, Laurent Riou, Loïc Djaileb, Clémence Charlon, Adrien Jankowski, Michele Magnesa, Caroline Augier, Stéphanie Marlière, Muriel Salvat, Charlotte Casset, Marion Maurin, Carole Saunier, Daniel Fagret, Catherine Ghezzi, Gerald Vanzetto, Gilles Barone-Rochette

**Affiliations:** 1 Department of Cardiology, University Hospital, 38000 Grenoble Alpes, France; lea.margerit@avec.fr (L.M.); imekhdoul@chu-grenoble.fr (I.M.); ccharlon@chu-grenoble.fr (C.C.); caugier@chu-grenoble.fr (C.A.); smarliere@chu-grenoble.fr (S.M.); msalvat@chu-grenoble.fr (M.S.); ccasset@chu-grenoble.fr (C.C.); mmaurin@chu-grenoble.fr (M.M.); csaunier@chu-grenoble.fr (C.S.); gvanzetto@chu-grenoble.fr (G.V.); gbarone@chu-grenoble.fr (G.B.-R.); 2 INSERM, U1039, Radiopharmaceutiques Biocliniques, Grenoble Alpes University, 38000 Grenoble Alpes, France; alexis.broisat@inserm.fr (A.B.); laurent.riou@univ-grenoble-alpes.fr (L.R.); ldjaileb@chu-grenoble.fr (L.D.); dfagret@chu-grenoble.fr (D.F.); catherine.ghezzi@univ-grenoble-alpes.fr (C.G.); 3 Department of Nuclear Medicine, University Hospital, 38000 Grenoble Alpes, France; 4 Department of Radiology, University Hospital, 38000 Grenoble Alpes, France; ajankowski@chu-grenoble.fr; 5 Department of Medical & Surgical Sciences, University of Foggia, 71121 Foggia, Italy; michele.magnesa17@gmail.com; 6 French Alliance Clinical Trial, French Clinical Research Infrastructure Network, 31059 Toulouse, France

**Keywords:** coronary atherosclerotic burden, dilated cardiomyopathy, cardiac magnetic resonance imaging

## Abstract

Background: Atherosclerosis is associated with a worse prognosis in many diseases such as ischemic cardiomyopathy, but its impact in non-ischemic dilated cardiomyopathy (dCMP) is lesser known. Our aim was to study the prognostic impact of coronary atherosclerotic burden (CAB) in patients with dCMP. Methods: Consecutive patients with dCMP and left ventricular (LV) dysfunction diagnosed by concomitant analysis of invasive coronary angiography (ICA) and CMR imaging were identified from registry-database. CAB was measured by Gensini score. The primary composite endpoint was the occurrence of major adverse cardiovascular events (MACE) defined as cardiovascular (CV) mortality, non-fatal MI and unplanned myocardial revascularization. The results of 139 patients constituting the prospective study population (mean age 59.4 ± 14.7 years old, 74% male), average LV ejection fraction was 31.1 ± 11.02%, median Gensini score was 0 (0–3), and mid-wall late gadolinium enhancement (LGE) was the most frequent LGE pattern (42%). Over a median follow-up of 2.8 years, 9% of patients presented MACE. Patients with MACE had significantly higher CAB compared to those who were free of events (0 (0–3) vs. 3.75 (2–15), *p* < 0.0001). CAB remained the significant predictor of MACE on multivariate logistic analysis (OR: 1.12, CI: 1.01–1.23, *p* = 0.02). Conclusion: High CAB may be a new prognostic factor in dCMP patients.

## 1. Introduction

The European Society of Cardiology (ESC) classification defines non-ischemic dilated cardiomyopathy (dCMP) as “the presence of left ventricular dilatation and left ventricular systolic dysfunction in the absence of abnormal loading conditions (hypertension, valve disease) or coronary artery disease (CAD) sufficient to cause global systolic impairment” [1].

However, CAD that is not sufficient to cause global systolic impairment, also known as bystander CAD, is frequent in dCMP patients. Indeed, bystander CAD is found in up to 30% of cardiac explants for the graft of patients with dCMP [2]. However, in these patients, such CAD is unlikely to be an innocent bystander. In the last decade, a large amount of data supported the prognostic role of coronary atherosclerotic burden (CAB), even beyond lumen stenosis quantification. Moreover, if several anatomic features may indicate vulnerable coronary plaques associated with an increased risk of adverse events in patients (e.g., noncalcified atherosclerotic plaques with positive remodeling, large lipid pool, speckled calcification, and other vulnerable characteristics) [3], none of these features has shown to be predictive of myocardial infarction and death independently of a comprehensive evaluation of coronary atherosclerotic disease burden. The risk continuum appears to be strongly associated with the burden of atherosclerotic disease [4,5]. Bart et al. showed that the angiographic extent of CAD was a stronger predictor of mortality in dCMP with ischemic versus nonischemic etiology [6]; however, this study has been performed without CMR data. Currently, it is well known that CMR is useful for the etiological diagnosis of dCMP [7]. Indeed, if invasive coronary angiography (ICA) is recommended to the etiological diagnosis of dCMP, particularly in patients with angina [8], the use of ICA alone may lead to a 13% incorrect assignment to dCMP caused by coronary recanalization after infarction [9]. Thus, the prognosis role of CAB is less well known in dCMP patients. The aim of this study was to evaluate the prognostic implication of CAB in patients with dCMP diagnosed with the systematic use of CMR and ICA.

## 2. Experimental Section

### 2.1. Study Population

From a prospective registry (ClinicalTrials.gov identifier: NCT03479580), we identified consecutive patients with dCMP who underwent CMR and ICA within one month between January 2014 and September 2016 in our institution. The gold standard for the diagnosis of dCMP was an expert consensus of a cardiomyopathy team composed of non-interventional and interventional cardiologists, radiologists, nuclear medicine physicians, internal medicine physicians and geneticists, established 3 months after admission, and based on data obtained during follow-up, including results of clinical, blood tests (serum calcaemia, phosphataemia, selenium, thiamine, carnitine, thyroid-stimulating hormone, serum protein electrophoresis, complete blood count, ferritin and transferrin saturation), and repeat imaging of new information, family history survey and *genetic tests.* The etiology of cardiomyopathy was considered ischemic or nonischemic on the basis of ICA-proven coronary artery disease according to Felker et al. [10] and on the basis of CMR according to Assomul et al. [7]. As described by Felker, patients were ascribed an underlying etiology of coronary artery disease (CAD) if there was an obstructive CAD with a stenosis >50% in the left main vessel or >75% either in the proximal left anterior descending artery (LAD) or at least two epicardial vessels. dCMP with bystander myocardial infarction presented global hypokinesia and small area of sub-endocardial late gadolinium enhancement (LGE) by CMR that did not explain the extent of left ventricular (LV) dysfunction and unobstructed coronary arteries on ICA. dCMP with a bystander CAD was defined by CMR showing no LGE or mid-wall LGE, global hypokinesia, and a significant CAD in ICA considered as insufficient to explain the extent of LV dysfunction (i.e., no stenosis >50% in the left main stem or no stenosis >50% with significant ischemia using fractional flow reserve (FFR) or single photon emission computed tomography (SPECT) in either the proximal LAD or at least two epicardial vessels other than LAD).

**Figure 1:**
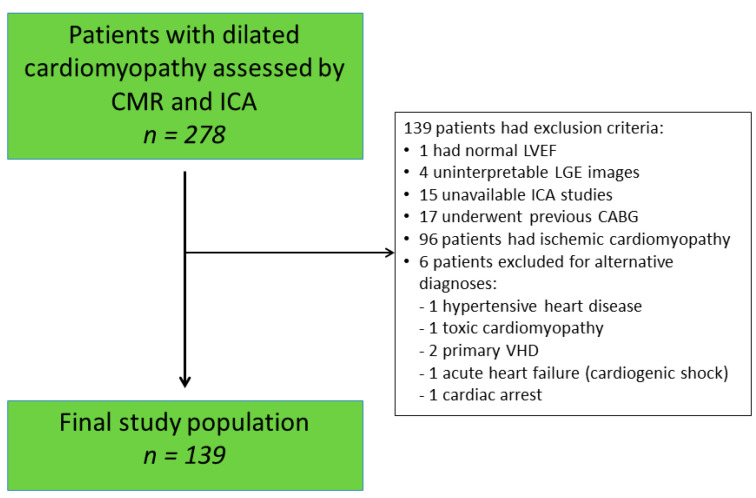
Flow chart of study. VHD: valvular heart disease. LVEF: left ventricular ejection fraction; CMR: cardiovascular magnetic resonance imaging; ICA: invasive coronary angiography; LGE: late gadolinium enhancement; CABG: coronary artery bypass grafting.

Exclusion criteria were patients with hemodynamic instability, with acute or chronic myocarditis, hypertrophic or hypertensive cardiomyopathy, severe valvular heart disease (VHD), arrythmogenic right ventricular cardiomyopathy, infiltrative cardiac disease, tachycardia-induced cardiomyopathy, cardiopathy secondary to toxins or alcohol abuse and congenital heart disease. Patients with previous revascularization (percutaneous coronary intervention (PCI) or coronary artery bypass grafting (CABG)) were excluded. If dCMP diagnosis remained ambiguous following expert committee analysis, the patient was excluded. Patients with contraindications to ICA or CMR such as claustrophobia, severe chronic kidney disease defined as a modification of diet in renal disease formula <30 mL/min/1.73 m^2^ and with a life expectancy < 1 year due to other comorbidities were also excluded. A total of 139 patients satisfied the inclusion criteria (Figure 1) and constituted the final study population. All participants provided written or oral informed consent for data evaluation and storage. The study conformed to the principles outlined in the Declaration of Helsinki and was approved by the ethical committee and institutional review board.

### 2.2. Data Collection

Patients’ medical history and treatment were prospectively recorded. Other information was retrieved from medical files and from the review of hospital records.

### 2.3. Cardiovascular Magnetic Resonance Imaging Protocol

CMR studies were acquired using 1.5-T or 3.0-T systems (Ingenia CV and Achieva, Philips Medical Systems, Best, the Netherlands) implemented with a 32-phase array, cardiac synergy surface coil. Cardiac synchronization was performed using a four-electrode vectogram. After a survey-scan, cine images were acquired using a steady-state free-precession (SSFP) breath-holding sequence (turbo fast echo) in standard two-, three-, and four-chamber long-axis views and subsequent 10 to 12 contiguous short-axis cines (30 phases/RR; breath-hold acquisition; slice thickness 8 mm without gap or 2 mm gap) from the atrio-venticular ring to the apex (shortest repetition time (TR)/echo time (TE) 2.8/1.4 msec; flip angle 45°; field of view (FOV) 320 nm; matrix 200 × 56). LGE images were acquired ten to fifteen minutes after intravenous injection of 0.2 mmol/kg gadolinium meglumine (Gd-DOTA) in identical short-axis planes using a breath-hold inversion-recovery gradient-echo T1-wheighted sequence (slice thickness of 14 mm, gap 0.7 mm, TR/TE 3.9/1.2 msec, flip angle 15°, FOV 320, matrix 185 × 256). Inversion times were adjusted in each patient, based on a look-locker sequence, to null the signal of normal myocardium. In the case of poor signal quality, a phase-sensitive inversion recovery (PSIR) sequence was acquired.

Ventricular volumes and mass and left atrial volume were calculated using the freely available software Segment version 2.2, Dublin, Ireland [11]. LGE by CMR was quantified with a fully automated method [12] as previously reported [13]. and the results were expressed as a percentage of myocardial mass. The distribution and pattern of LGE was assessed by 2 independent observers who were blinded to the clinical data, coronary anatomy, and outcomes and with over 10 years of experience (EACVI level 3). Discordant findings were resolved by consensus.

### 2.4. Invasive Coronary Angiography

Selective conventional ICA was performed using standard techniques (Philips Allura Xper FD10, Philips Healthcare, Suresnes, France). All patients were evaluated using quantitative coronary angiography (QCA (Vepro Computer systeme GmbH, Pfungstadt, Germany)) by one experimented cardiologist (G.B.) blinded to the CMR results. Intermediate stenosis defined by a reduction in vessel diameter between 50% and 70% were assessed for significant ischemia using fractional flow reserve (FFR) or single photon emission computed tomography (SPECT) according to international recommendations and as previously published [14]. CAB was determined by the Gensini score. In brief, atherosclerotic lesions with 25%, 50%, 75%, 90%, 99%, and 100% lumen obstruction were angiographically quantified and scored with 1 (25% stenosis), 2 (50% stenosis), 4 (75% stenosis), 8 (90% stenosis), 16 (99% stenosis), and 32 (100% stenosis, total obstruction) points, respectively. Afterward, a multiplying factor (from 1 to 5) was used depending on the significance of the area supplied by a given coronary segment in which the lesion resides. The final coronary artery atherosclerotic score was the sum of all segment scores [15]. Gensini score is a widely used means of quantifying angiographic atherosclerosis, where a zero score indicates absence of atherosclerotic disease. The median (interquartile range) of the Gensini score depends then on the risk level of the study population.

### 2.5. Follow Up and Endpoints

Patients were prospectively followed until June 2019. A self-administered questionnaire was sent, and non-responders were contacted by telephone interviews. If they still did not respond, their cardiologists or general practitioners were reached. The duration of follow-up was calculated from baseline CMR scan until an endpoint occurred or last patient contact. All events were adjudicated by two operators (G.B., M.C.) who reviewed electronic databases and documents of hospitalization or medical procedure. The primary endpoint was a composite of CV death, non-fatal MI [16], unplanned myocardial revascularization by CABG or percutaneous coronary intervention (PCI) at least 3 months after enrollment. CV death was defined as death due to acute MI, sudden death (i.e., unexpected, unwitnessed, or witnessed death in absence of other apparent causes), or death due to HF, stroke or following a CV procedure.

### 2.6. Statistical Analysis

Statistical analyses were performed using SPSS version 24 (IBM SPSS Statistics, IBM Corporation, Armonk, New York, NY, USA) software. Data are presented as mean ± SD, median (interquartile range), or number (%) of patients. Differences in normally distributed continuous variables were tested by an unpaired Student’s t test. The Mann–Whitney U test was used to compare abnormally distributed continuous variables. Categorical data were compared with a χ2 test or the Fisher exact test where appropriate. Logistic regression analysis was performed to establish the predictors of all-cause mortality. Variables that were significant in univariate analysis were then used in multivariate logistic regression analysis. The odds ratio (OR) and 95% confidence interval (CI) were calculated for each independent variable. The dichotomous Gensini score were chosen based on the Youden J index results and cumulative events curves were constructed for time to event using the Kaplan–Meier methods and tested for significance using log-rank statistics. All tests were 2-sided, and *p*-value < 0.05 was considered statistically significant.

## 3. Results

### 3.1. Study Population

**Figure 2:**
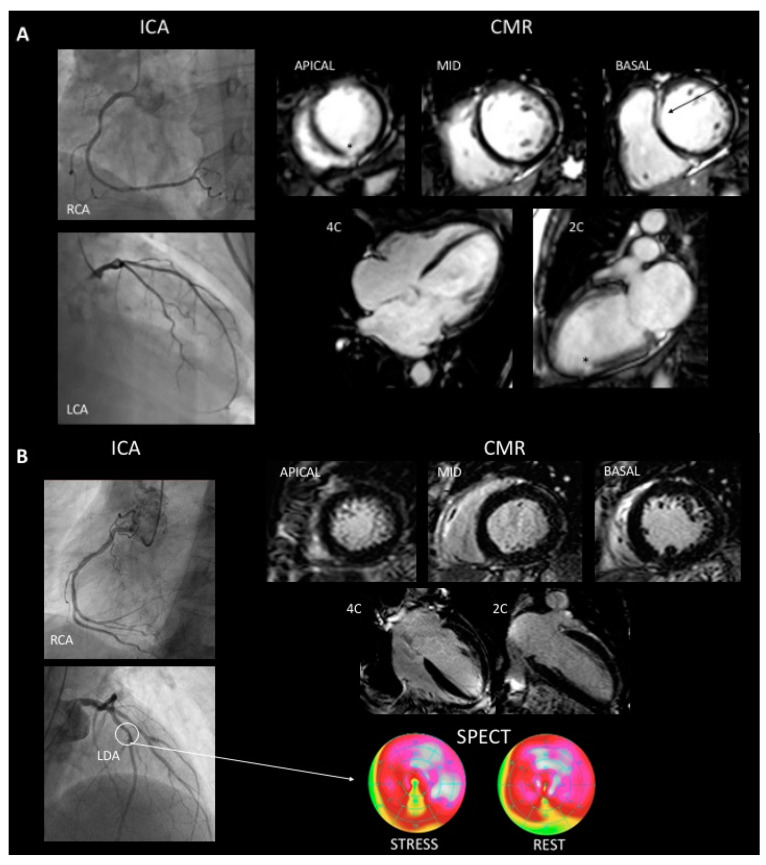
A 66-year-old woman who presented withg dCMP and bystander myocardial infarction. ICA was normal and CMR showed a 31% LVEF, an end-diastolic volume of 131 mL/m^2^, global hypokinesia and 2 patterns of LGE. Mid-wall linear septal (arrow) and small sub-endocardial LGE (asterisk) (Panel **A**). A 71-year-old man who presented dCMP with coronary artery disease. ICA shows plaque to 40–50% on left descending anterior artery (arrow), CMR showed a 32% LVEF, an end-diastolic volume of 123 mL/m^2^, global hypokinesia and no LGE (Panel **B**). LCA: Left coronary artery, LDA: left descending artery, RCA: right coronary artery, ICA: invasive coronary angiogram, CMR: cardiac magnetic resonance imaging, SPECT: single photon emission computed tomography.

The mean age of patients was 59.4 ± 14.7 years, 74% were men and the LVEF was of 31.1 ± 11.02%. Table 1 summarizes patients’ baseline characteristics. The mean body mass index (BMI) was 26.25 ± 4.79, 42% of patients were tobacco smokers, 22% had diabetes mellitus, 35% high blood pressure and 31% dyslipidemia. Ninety-two patients (66%) had LGE on CMR images. The most common fibrosis pattern was mid-wall in 42% of patients, followed by sub-endocardial (15%) and multiple patterns (9%). Focal LGE was found in two patients (2%). The mean Gensini score was 0 (0–3). In the cohort, 101 patients (73%) were classified as true dCMP, 30 patients (22%) presented dCMP with bystander MI, and 8 patients (5%) presented dCMP with bystander CAD. Significant ischemia of bystander CAD was ruled out by SPECT in all cases. Figure 2 shows multimodal imaging assessment of patients with dCMP with bystander MI (panel A) and patient with dCMP with bystander CAD (panel B).

### 3.2. Events at Follow-Up

A total of 12 patients (8.63%) reached primary outcome after a median follow-up of 2.8 years, and seven patients died of CV causes. Two patients died of terminal HF and five patients suffered sudden cardiac death. One patient presented a MI with pPCI and four patients underwent coronary artery revascularization by PCI for worsening HF and ischemia proven by SPECT (two cases) or FFR (two cases). For these patients, baseline and follow-up data were reviewed, to determine if PCI was performed for a known or a new coronary lesion. Two patients received PCI for novel coronary artery lesions and the two others for the progression of known coronary stenoses. The basal characteristics of patients are presented in Table 1. There was no difference in gender among patients that presented events as compared to others (83% male vs. 73%, *p* = 0.4), but patients with MACE at follow-up were more likely to be older (70 ± 8.89 vs. 58.4 ± 14.7 years, *p* = 0.008), to have diabetes (50% vs. 19% *p* = 0.02), and dyslipidemia (58% vs. 29%, *p* = 0.051). In patients without MACE at follow-up, LVEF was higher, as compared to patients that presented MACE (31.7 ± 11.1% vs. 24.1 ± 6.64%, *p* = 0.02). Similarly, patients without MACE had a better right ventricular ejection fraction (RVEF) (42.9 ± 14.1% vs. 32.8 ± 10.3%, *p* = 0.004). The presence, extent, localization, and pattern of LGE were not statistically significant between MACE or no MACE patients. Patients with MACE had a higher Gensini score than patients without MACE (0 (0–3) vs. 3.75 (2–15), *p* < 0.0001).

### 3.3. Predictors of Events

**Figure 3:**
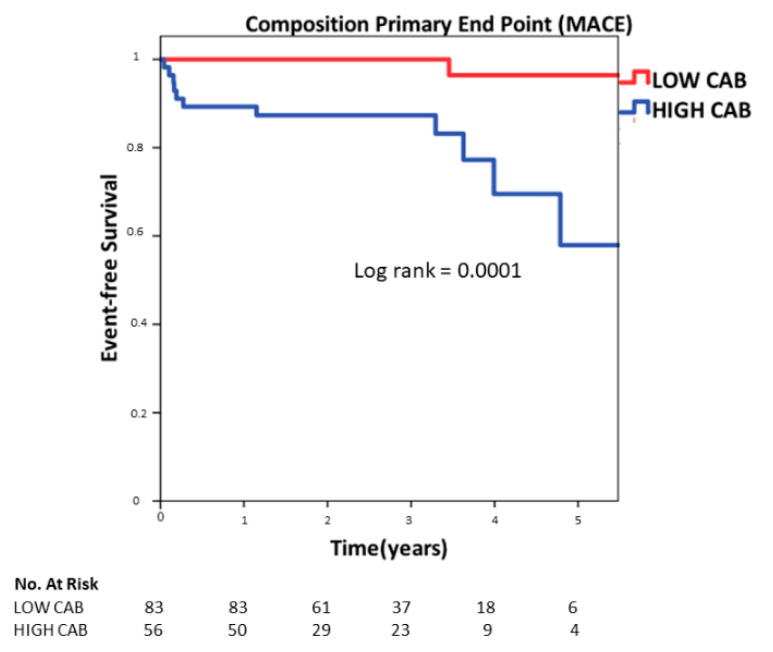
Kaplan–Meier event-free survival curve for the composite endpoint of cardiovascular death, non-fatal myocardial infarction (MI) or coronary artery revascularization, stratified by high or low CAB.

**Figure 4:**
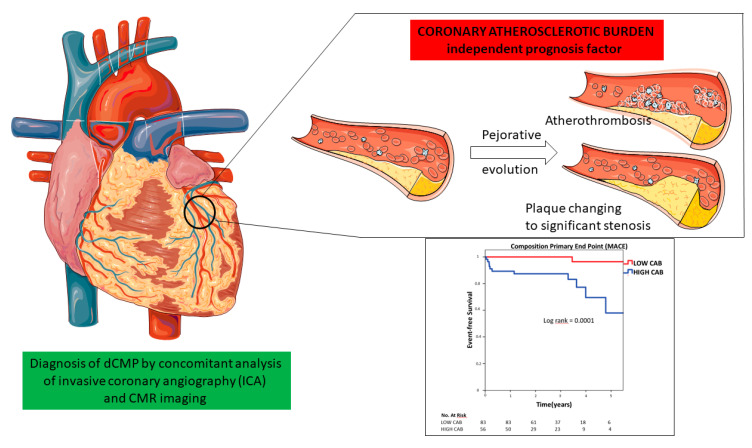
Central illustration: coronary atherosclerotic burden and dilated cardiomyopathy prognosis. CMR: cardiovascular magnetic resonance imaging.

Univariate analysis to establish the possible predictors for MACE identified age (OR: 1.08, 95% CI: 1.01–1.15, *p* = 0.01), diabetes (OR 4.16, 95% CI: 1.23–14.05, *p* = 0.02), dyslipidemia (OR 3.42, 95% CI: 1.01–11.4, *p* = 0.04), LVEF (OR 0.93, 95% CI: 0.88–0.99, *p* = 0.02), LVESVi (mL/m^2^) (OR: 1.01, 95% CI: 1.00–1.03, *p* = 0.04) and CAB (OR: 1.10, 95% CI: 1.02–1.18, *p* = 0.009) as the independent predictors of MACE (Table 2). Multivariate nominal logistic regression including the predictors identified on univariate analysis identified age (OR: 1.09, 95% CI: 1.01–1.17, *p* = 0.02), and CAD (OR 1.12, 95% CI:1.01–1.23, *p* = 0.02) as the only independent predictors for all-cause mortality as shows in Table 3. Figure 3 shows the Kaplan–Meir survival plot showing elevated CAB greater than the Gensini score >1.25 to be associated with increased MACE at long-term follow-up (*p* = 0.0001 by log rank test) (Figure 4).

## 4. Discussion

This is the first study to date to examine the prognostic role of CAB evaluated by ICA in a large, well-phenotyped dCMP cohort by the systematic use of CMR and ICA. Our study shows that the detection of high CAB by Gensini score can predict MACE in dCMP patients. To date, no study has directly addressed this question. 

The results are concordant with studies demonstrating that increasing CAB constitutes a risk of adverse events in other populations. Numerous clinical studies using conventional invasive such as ICA [17], IVUS [18], coronary artery calcium (CAC) score [19], and cardiac computed tomography (CTA) [20,21] have confirmed the strong relationship between CAB and MACE in the general population. This imaging strategy appears to be better than the identification of cardiovascular risk factors. Lin FY et al. have shown that even among patients with a low Framingham risk score, the extent of nonobstructive coronary artery disease was associated with an increased risk of mortality [22]. Evaluating the extent of nonobstructive coronary artery disease has the advantage of focusing on outcomes that are associated with the actual disease process detected by CAB imaging. Even if the low number of events represents a limitation to the present study, we observed that Gensini score was predictive of cardiac events independently from risk factors. Gensini score is one of the multiple scoring systems devised to quantify angiographic CAB [23]. While CAB could be underestimated using ICA, because it only reflects the reduction in luminal area and does not visualize the whole plaque, Gensini score correlated significantly with the average plaque burden and plaque area, as determined by IVUS (0.76, *p* < 0.0001) [23]. If IVUS is clearly more efficient to provide information concerning the integrity of the arterial wall and detect vulnerable plaque [24], it is less invasive, in order to obtain global CAB, to use quantified angiographic CAB. Moreover, if several anatomic features may indicate vulnerable coronary plaque which increase the risk of adverse events in patients, none of these features have shown to be predictive of myocardial infarction and death independently of a comprehensive evaluation of CAB. The risk continuum appears to be strongly associated with the burden of atherosclerotic disease [4,5]. In a recent study, Mortensen et al. showed again that plaque burden, not stenosis per se, is the main predictor of risk for CVD events and death in patients with coronary atherosclerosis [25].

Four patients presented late nonurgent revascularizations. However, we observed that the increased rate of interventions in this group occurred mainly during follow-up (median follow up at 3.4 years), possibly when those patients experienced the progression of their cardiac disease. Indeed, the PARADIGM (Progression of AtheRosclerotic PlAque DetermIned by Computed TomoGraphic Angiography IMaging) study suggested the early capability of plaque volume analysis to identify patients with a higher risk of plaque progression, even in the absence of significative coronary stenosis at baseline [26]. The progression disease in dCMP patients is significant to consider. Indeed, studies have shown that CAD and dCMP could coexist at baseline [27] and could progress over time in dCMP [2], leading to unplanned hospitalizations [28]. In our study population, the Gensini score was relatively low. Indeed, as a comparison, in a recent study which prospectively enrolled 2149 consecutive patients who underwent first coronary angiography with a history of any type of chest pain and without dCMP, the patients were classified into three groups according to tertiles of Gensini score: low Gensini acore (<5), intermediate Gensini score (5–33), and high Gensini score (>34) [29]. Therefore, in spite of low Gensini scores in our study, we identified patients with a high or low CAG and we demonstrated that CAB predicts ischemic CV events and that high-risk patients could benefit from closer follow-up. This finding is in accordance with that of Repetto et al. [2], suggesting that ICA must be repeated even sooner for unexpected, unexplained decompensated heart failure in dCMP patients whose initial diagnostic studies demonstrated coronary atherosclerotic plaques, albeit non-occlusive at the time. Assessing this parameter improves risk-stratification for future ischemic events and could be used to help personalize preventive therapy by antiplatelet and lipid-lowering therapies in this population [30]. Assessments provide powerful prognostic information; however, nowadays neither invasive or noninvasive evaluation of atherosclerotic burden for prognostic stratification has any role in clinical cardiovascular guidelines.

Frankenstein et al. [31] followed 1263 patients with dCMP, 32.9% of whom had a bystander obstructive CAD that was not predictive of all-cause mortality or re-hospitalization for HF. However, there was no CMR data in their study and patients could be misclassified. Additionally, coronary atherosclerosis burden was not quantified. Finally, they studied the impact of obstructive CAD on a HF endpoint, while in our study, MACE was selected as the endpoint.

Another predictive factor of ischemic events in our study was LVEF. This is consistent with Repetto et al. [2], who suggested that inflammation and neuro-hormonal responses in HF with reduced EF are associated with higher susceptibility in atherogenic and thrombotic events, hence to the development of stable and unstable coronary events. Age, and diabetes were also associated with future ischemic events in our study. It is not surprising, as they are some of the main CV risk factors.

Mid-wall fibrosis was the predominant LGE pattern in our dCMP patients and it did not predict ischemic events. Gulati et al. [32] showed that mid-wall fibrosis is associated with all-cause mortality, HF and arrhythmic endpoints in this population, but its impact on future ischemic events was never analyzed. Histology studies demonstrated that mid-wall LGE is associated with replacement fibrosis that follows myocyte death. Although the underlying mechanisms are not fully understood, mid-wall fibrosis in our cohort of patients does not reflect CAB [33].

CMR and ICA data appeared crucial to make a precise diagnosis of dCMP; moreover, CAB by Gensini score may be an independent prognosis factor of MACE. However, the calculation of Gensini score is time consuming and is not widely used in clinical practice. Today, the field of medical imaging analysis presents dazzling progress thanks to artificial intelligence (AI). Deep learning is probably the most powerful form of AI for automated image analysis today [34]. Made up of a network of artificial neurons, it allows, using a very large number of known examples, one to extract the most relevant characteristics of the image to solve a given problem. Deep learning networks accurately identified and segmented the major vessels in X-ray coronary angiography and by applying deep learning segmentation, CAB analysis could be further automated, thereby facilitating the use of Gensini methods [35].

### Limitation

This study was performed in a single center. Although it was an experienced center and many patients were referred from peripheral centers, it can trigger potential bias. However, patients’ baseline characteristics were similar to other dCMP registries [32,36]. The etiological diagnosis of cardiomyopathy and the bystander character of CAD or infarction may be difficult to determine in certain cases of dCMP, although the expert consensus of a cardiomyopathy team used the last recommendations [37,38,39]; moreover, unclear cases were excluded. Ultrasound data analysis with post treatment was not disponible. Adjunctive tests of ICA including guidewire-based techniques with adenosine and coronary reactivity testing, which are recommended now to a complete exploration of CAD, were not used [40,41]. Large-scale observations showed that despite recommendations, women are treated less aggressively, with fewer cardiac catheterizations [42]. This certainly explains the under-representation of women in our study, which is another limitation. The survival analysis included in this study was based on a relatively low number of cardiac events recorded during follow-up and should be considered of speculative interest. Its statistic power was limited by a modest number of events.

## 5. Conclusions

Our study establishes that CAB may be an independent predictor of MACE in patients with dCMP. This supports the systematic evaluation of CAB by ICA for risk stratification in dCMP patients after the systematic use of CMR and ICA for the etiological diagnosis of cardiomyopathy.

## Figures and Tables

**Table 1:** Baseline characteristics.

	All Patients (*n* = 139) 100%	MACE (*n* = 12) 9%	No MACE (*n* = 127) 91%	*p*-Value
Age, years	59.4 (14.7)	70.0 (8.89)	58.4 (14.7)	0.008
Male	103 (74)	10 (83)	93 (73)	0.4
BMI, kg/m^2^	26.25 (4.79)	27.0 (5.70)	26.1 (4.70)	0.5
Ever smoker	58 (42)	4 (33)	54 (44)	0.4
Diabetes mellitus	30 (22)	6 (50)	24 (19)	0.02
Dyslipidemia	43 (31)	7 (58)	36 (29)	0.051
Hypertension	48 (35)	5 (41)	43 (34)	0.6
Family history of CAD	20 (14)	1 (8)	19 (15)	0.5
CMR measurements				
LVEF (%)	31.1(11.02)	24.1 (6.64)	31.7 (11.1)	0.02
LVEDVi (mL/m^2^)	123.5 (35.4)	139.1 (23.0)	120.0 (36.0)	0.1
LVESVi (mL/m^2^)	86.3 (34.6)	106.0 (22.4)	84.0 (35.1)	0.03
LV mass index (g/m^2^)	72.4 (20.6)	78.5 (19.5)	71.8 (20.7)	0.2
LA size (mL/m^2^)	72.9 (25.5)	80.8 (24.9)	72.2 (25.6)	0.3
Cardiac index (L/mn/m^2^)	2.38 (0.72)	2.73 (0.94)	2.34 (0.69)	0.08
RVEF (%)	41.8 (14.3)	32.8 (10.3)	42.9 (14.1)	0.04
RVEDVi (mL/m^2^)	91.8 (32.2)	94.4 (28.6)	91.5 (32.9)	0.8
RVESVi (mL/m^2^)	54.2 (29.0)	65.2 (29.1)	52.3 (28.9)	0.2
LGE				
Presence (%)	93 (66)	9 (75)	84 (66)	0.5
Extent (% of total LV mass)	7.4 (10.03)	9.63 (10.88)	7.19 (9.9)	0.4
LGE by Location				
LGE septal (%)	57 (41)	7 (58)	50 (39)	0.2
LGE by pattern and distribution				
-Sub-endocardial (%)	21 (15)	3 (25)	18 (14)	0.39
-Mid-wall linear (%)	58 (42)	4 (33)	54 (43)	0.53
-Mid-wall nodular (%)	2 (1)	0 (0)	2 (2)	0.8
-Multiple patterns (%)	12 (9)	2 (16)	10 (8)	0.27
Coronary atherosclerotic burden				
Gensini score	0 (0–3)	3.75 (2–15)	0 (0–3)	0.0001

Values are mean ± standard deviation (SD) or median (interquartile range) or *n* (%). BMI: body mass index; CAD: coronary artery disease; CMR: cardiovascular magnetic resonance imaging; LA: left atrium; LGE: late gadolinium enhancement; LV: left ventricular; LVEDVi: left ventricular end-diastolic volume index; LVEF: left ventricular ejection fraction; LVESVi: left ventricular end-systolic volume index; RVEF: right ventricular ejection fraction; RVEDVi: right ventricular end-diastolic volume index; RVESVi: right ventricular end-systolic volume index; PCI: percutaneous coronary intervention.

**Table 2:** Univariate logistic regression analysis for MACE.

	OR	95% CI	*p*-Value
Age, years	1.08	1.01–1.15	0.01
Male	0.54	0.11–2.62	0.45
BMI, kg/m^2^	1.03	0.92–1.17	0.54
Ever smoker	1.56	0.44–5.47	0.48
Diabetes mellitus	4.16	1.23–14.05	0.02
Dyslipidemia	3.42	1.01–11.4	0.04
Hypertension	1.36	0.40–4.54	0.61
Family history of CAD	0.49	0.06–4.08	0.51
CMR measurements			
LVEF (%)	0.93	0.88–0.99	0.02
LVEDVi (mL/m^2^)	1.01	0.99–1.02	0.11
LVESVi (mL/m^2^)	1.01	1.00–1.03	0.04
LV mass index (g/m^2^)	1.01	0.98–1.04	0.28
LA size (mL/m^2^)	1.01	0.98–1.03	0.33
Cardiac index (L/mn/m^2^)	1.95	0.90–4.21	0.08
RVEF (%)	0.94	0.89–1.00	0.054
RVEDVi (mL/m^2^)	1.00	0.98–1.02	0.80
RVESVi (mL/m^2^)	1.01	0.98–1.03	0.23
LGE			
Presence (%)	1.53	0.39–5.96	0.53
Extent (% of total LV mass)	1.02	0.97–1.07	0.42
LGE localization			
LGE (septal)	2.15	0.64–7.16	0.21
LGE by pattern and distribution			
-Sub-endocardial (%)	2.01	0.49–8.17	0.32
-Mid-wall linear (%)	0.67	0.19–2.36	0.53
-Mid-wall nodular (%)	–	–	–
-multiple patterns (%)	2.34	0.44–12.1	0.31
Coronary atherosclerotic burden			
Gensini score	1.10	1.02–1.18	0.009

**Table 3:** Multivariate logistic regression analysis for MACE.

	**OR**	**95% CI**	***p*** **-Value**
Age, years	1.09	1.01–1.17	0.02
Diabetes mellitus	1.85	0.32–10.5	0.48
Dyslipidemia	2.15	0.41–11.03	0.35
LVEF (%)	0.91	0.79–1.03	0.16
LVESVi (mL/m^2^)	1.00	0.97–1.04	0.73
Gensini score	1.12	1.01–1.23	0.02

## Data Availability

The data presented in this study are available on request from the corresponding author. The data are not publicly available due to ethical and privacy restrictions (DRCI CHU de Grenoble).
